# Research on the Circumstances of a Car–Cyclist Collision, Based on the Trajectory of the Cyclist’s Movement after the Collision

**DOI:** 10.3390/s22176324

**Published:** 2022-08-23

**Authors:** Edgar Sokolovskij, Edvinas Juodka

**Affiliations:** Department of Automobile Engineering, Faculty of Transport Engineering, Vilnius Gediminas Technical University, J. Basanaviciaus str. 28B, LT-03224 Vilnius, Lithuania

**Keywords:** traffic accident, car–cyclist collision, accident modeling, throwing distance, throwing angle

## Abstract

This article examines a simulated collision between a car and a cyclist, assessing the trajectory of the cyclist’s movement after the impact, namely the throwing distances and angles of the cyclist and bicycle. Information about the car and cyclist models used for the study is provided. Special software PC CRASH 8.1 for the analysis and reconstruction of traffic accidents was used to simulate a car–cyclist collision. Simulations of car–cyclist collisions were carried out, with different speeds for the car and the cyclist, and locations at the time of the impact. The movement of a bicycle after a crash tends to be irregular and is dependent on various parameters that are usually not possible to evaluate. Therefore, the parameters of the movement of the bicycle after the collision (the throwing angle and the distance) usually do not allow determination of the speed of the car before the accident. The movement of the cyclist after impact was more informative for determining the speed of the car before the accident. For example, when there was an angle of 30°, 60°, or 90° between the longitudinal axes of the car and the cyclist, there was a clear dependence between the speed of the car and the cyclist’s throwing distance, and usually also between the speed of the car and the cyclist’s throwing angle. Thus, in such cases, it is possible to determine approximately the initial speed of the car before the collision, based on the trajectory of the cyclist’s movement after the impact, namely his throwing distance and angle. In cases of real traffic accidents, with knowledge of the location of the car–cyclist collision and the position of the cyclist after the traffic accident, the speed of the car before the accident can be determined according to the abovementioned dependencies. Thus, the proposed methodology could be used in the reconstruction and examination of traffic accidents.

## 1. Introduction

### 1.1. Literature Review

Accidents often affect not only vehicles but also people [1]. One such type of accident, frequently characterized by major injuries to the accident participant, is the hitting of a cyclist. This is because, as when a pedestrian is hit, the cyclist is comparatively less protected from injury. Injuries can range from very mild to very severe, and fatalities are common. Depending on the circumstances and consequences of the accident, disagreements may arise about the cause of the accident, the identification of the perpetrator, or other details. In order to understand the circumstances of a traffic accident, accident examinations are performed, which assess such factors as the speed of vehicles, directions of movement, and traffic conditions. Research methodologies for traffic accidents such as bicycle crashes need to be improved, and a number of aspects require further investigation.

In 2020, a total of 3022 traffic accidents were registered in Lithuania, of which 246 were caused by drunk drivers [2]. Between 2016 and 2019, a total of 51 cyclists were killed and 1109 injured. In these accidents, cyclists accounted for an average of 4.7% of all perpetrators. According to Lithuanian statistics, traffic accidents occur due to reasons such as speeding, unsafe overtaking, poor assessment of traffic conditions, non-compliance with road traffic rules, and lack of traffic culture. The consumption of alcohol and psychotropic substances as well as fatigue can also have a negative impact [3].

Globally, traffic accidents involving cyclists are mostly frequent in China, where bicycles are the most commonly used means of transportation. As an example, about 77% of vehicles in Tianjin are bicycles, while in Sydney, Australia, bicycles account for only 1% of all vehicles. It is estimated that one in four people in China has a bicycle. Although cyclists account for 45% of all fatalities in accidents, the use of helmets there is rare [4].

In the event of an accident, it is necessary to discover the reasons why it occurred. The most common reasons relate to the driver, the vehicle, and the road. Accident analysis is intended to identify information about accident participants, vehicles, road conditions, and their influence on the occurrence of the accident [5].

Many scientists have studied the influence of different factors on the occurrence of traffic accidents [6,7,8], issues of accident reconstruction [9,10,11], and the use of various parameters in reconstruction performance [12]. In the event of a cyclist being hit, as in other traffic accidents, it is very important to accurately represent the course of the accident.

Cases of hitting a cyclist have been examined in scientific publications to assess cyclist injuries during such events. Veisten et al. [13], for example, considered injuries to cyclists in road accidents in Norway. Injuries to individual parts of the body were identified by Raslavičius et al. [14]; four event scenarios were modeled for a car crashing into a cyclist from different angles. It was found that the speed of the car and the position of the vehicles before the collision had the greatest influence on the extent of bodily injury. According to the generalized injury scale, the cyclist suffered injuries from level 3 to level 6.

An article by Katsuhara et al. [15] considered the kinematic regularities of impacts, where in one case a cyclist was hit by an sport utility vehicle (SUV), and in anotherby a sedan-type car. Accident simulations were developed on the basis of finite elements. When the cyclist came in contact with the sedan-type car, the pelvis of a the person hit slipped on the engine cover until their head hit the windshield of the car. In the case of the SUV, the cyclist’s pelvis suffered an impact on the front of the engine cover, which restricted the movement of the body. Restriction of body movement reduced the cyclist’s head displacement.

Pang et al. [16] investigated three variants of traffic accidents. Three accident scenarios were modeled with MADYMO software. In the first variant, the cyclist lost control of the bicycle at 35 km/h and collided with a metal barrier. Their body was thrown to the opposite side of the barrier. In the second case, the cyclist was hit by a car traveling at 75 km/h from the right-hand side. The third case described a collision between two cyclists, after which one cyclist collided with a roadside barrier. Referring to the second case, when the cyclist was moving at a speed of 10 km/h, the right leg made contact, after which the body slipped on the engine cover until the head hit the lower part of the windshield. When the cyclist’s speed was increased to 20 km/h, the head did not make contact with the car windshield.

An article by Cicchino et al. [17] analyzed accidents involving electric scooters. In 2019, 105 teenagers involved in traffic accidents were interviewed. The survey found that the teenagers surveyed suffered more severe injuries than moderate (>AIS 2). It is also likely that the higher the speed of the electric scooter, the more serious were the injuries suffered.

An article by Isaksson-Hellman and Werneke [18] described the scenarios and frequencies of traffic accidents, using detailed information on accidents involving cars and cyclists in Sweden, including 882 accident reports received for analysis. The reports highlighted particular factors before and during the accident. Factors before the accident were described by variables such as the position of the driver and the cyclist in relation to each other, the speed of the motor vehicle before the accident, and the level of visibility. The accident itself was described by indicating the place and direction in which the vehicles collided, as well as parameters of braking, wheel turning, environmental conditions, and the place of collision. The most common scenario was where one vehicle crossed the direction of movement of another vehicle (78.1%). The second scenario in terms of frequency was that both vehicles were moving in the same or opposite directions (10.7%), and meanwhile 4.4% of accidents were due to a cyclist hitting a vehicle door. Other accidents accounted for 3.4%. Injuries to road users were assessed using the generalized injury scale (AIS), an anatomical coding system that divides injuries into six levels.

The kinematics of the cyclist’s movement and his injuries during the crash were examined in an article by Nie and Yang [19]. They also analyzed the mechanism of throwing a cyclist onto the engine cover of a vehicle, and the factors influencing this.

When a car hits a cyclist, contact points appear on the car body, from which the distance to the ground is measured according to the shape of the car. The measured distance is called the WAD parameter, described as the distance from the contact of the human head with the car to the road surface at the front of the car. The article by Yuan [20] examined the dependence of various parameters on the WAD parameter when a pedestrian is hit.

### 1.2. Research Motivation

After analyzing the literature sources, it can be seen that in cases of car–cyclist collisions, it is usually not possible to calculate the initial speed of the car before the accident by applying methods used in the analysis of car collisions, such as the law of conservation of momentum, etc. This is because the mass of the car and the cyclist are very different, and such a calculation would be very inaccurate. In this case, it is also difficult to determine the speed of the car before the traffic accident by simulating one specific individual accident. Exact simulation of a car–cyclist collision, fully re-creating the course of the event and the movement of vehicles after the collision, is problematic due to the specifics of the bicycle’s movement.

In the case of a car–pedestrian collision, expert studies have sometimes used the method of determining the approximate speed of the car before the accident based on the distance the pedestrian’s body was thrown after the collision. It would be appropriate to create a similar methodology in cases of car–cyclist collision. However, car–cyclist collision cases have multiple specifics. In such cases, there is not only a cyclist but also a bicycle involved; it is therefore a multibody system, with a different nature of contact, and a different position of the human body in relation to the car than in the case of a pedestrian.

Therefore, this study aims to determine whether there is a relationship between the initial speed of the car and the throwing distances of the cyclist and the bicycle after the accident, in cases of car–cyclist collision at different collision angles. The dependence on the cyclist and the bicycle’s throwing angle after the collision was also analyzed. In cases of real traffic accidents, the location of the initial contact as well as the final positions of the cyclist and the bicycle are usually known or can be determined after the traffic accident. It is therefore appropriate to examine the full throwing distance from the place of initial contact with the car to the final position after the accident. After determining that such a dependence exists, it would be possible to refine this methodology and perform further simulations under different road and collision conditions. The use of the summarized results of such simulations would allow experts investigating car–cyclist collision cases, knowing the throwing distance and the throwing angle of the cyclist and the bicycle after the traffic accident, to determine the speed of the car before imapact and perform a more accurate reconstruction of the traffic accident.

The subject of this research is a cyclist and his movement after a collision with a car. The analysis of the cyclist’s collisions and the computer modeling were designed to determine the relationship between the speed of the car before the accident and the distance and angle at which the cyclist was thrown. This should allow more accurate investigation of the circumstances of cycling crashes during accident examination.

## 2. Theoretical Analysis and Reconstruction of a Cyclist’s Collision, Models

Bourdet et al. [21] analyzed reconstructions of traffic accidents involving car and bicycle collisions. Reconstruction of each event was performed using MADYMO software, and eight accidents were reconstructed using a model developed by the French Institute (IFSTTAR). Another 18 accidents were reconstructed using the Unistra TNO model. The simulation of the finite element method was performed using RADIOSS software. The car model, developed by the French Institute (IFSTTAR), consisted of circular and elliptical bodies.

During the reconstruction of the traffic accident, the speeds of the car and the cyclist were determined just before impact. The above-mentioned parameters can be determined based on the principle of energy transfer between the car and the cyclist, or on the stopping distance. The main aspects of the reconstruction methodology were as follows:

- The test results depended on such unknown parameters as height, weight, vehicle speed, stopping distance.
- During the simulation of the event, the cyclist, the bicycle and the car were linked.
- The adjustment of the event reconstruction was performed by comparing the initial known parameters with the parameters obtained after the simulation.

An article by Condrea et al. [22] dealt with the model when a cyclist is hit by a car on the rear wheel of the bicycle. During the initial impact, a force of impact occurs between the wheel of the bicycle and the bumper of the car. The impact force also generates forces that act on the contact between the cyclist and the bicycle seat.

Fitton and Symons [23] examined the model of a cyclist moving on a velodrome. The kinetic energy of the system is described by the following expression:

(1) $$T=2\left(\frac{1}{2}m_{w}v_{cw}^{2}\right)+\frac{1}{2}m_{c}v_{c}^{2}+2\left(\frac{1}{2}I_{w}\omega_{w}^{2}\right)+\frac{1}{2}I_{c}\omega_{c}^{2};$$

where *m_w_* is mass of one bicycle wheel; *v_cw_* is linear wheel speed; *m_c_* is the mass of the cyclist/bicycle; *v_c_* is the cyclist/bicycle linear speed; *I_w_* is the moment of inertia of one bicycle wheel; *ω_w_* is angular wheel speed; *I_c_* is the moment of inertia of the cyclist/bicycle; *ω_c_* is cyclist/bicycle angular speed.

The hitting of a cyclist can in principle be examined in the same way as the hitting of a pedestrian [24]. The course of such events can be divided into three stages:

1. During a car–cyclist collision, the impulse of the impact is transmitted from the car to the cyclist. When the contact between the car and the cyclist breaks, this stage is complete.
2. Depending on the configuration of the collision, a period ensues during which the cyclist is thrown, until the first contact between the cyclist and the ground occurs.
3. When the first contact between the cyclist and the ground occurs, the victim slips or turns until he stops completely.

Of course, the hitting of a cyclist has its own specificity only because it is a system of masses consisting of individual elements, i.e., the bicycle and the human body, and other determining factors, namely the location of the center of weight. There is also the specificity of primary contact; in the event of the hitting of a cyclist, the primary contact is usually between the front of the car and the bicycle, rather than directly with the human body as is the case with pedestrian collision.

The target is to obtain the total throwing distance as a function of the car’s initial speed. This principle can be applied in the event of a cyclist being thrown. Considering that the cyclist is a mass system consisting of a bicycle and a person, as already mentioned, and the speed of a cyclist is slightly higher than that of a pedestrian, it is therefore appropriate to examine the throwing distances of both the bicycle and the cyclist, as well as the angles at which the bicycle and the cyclist are thrown, and their dependence of the speed of the car.

The initial speed of the cyclist is described by the following expression:

(2) $$v_{c0}=kv_{a0}^{\prime };$$

where *k* is constant size; $v_{a0}^{\prime }$ is the speed of the car after the cyclist is hit.

During the collision, the car transmits the impact impulse to the cyclist according to the law of endurance of the amount of motion:

(3) $$v_{a0}^{\prime }=\frac{m_{a}}{m_{a}+m_{c}}v_{a0};$$

where *v_a_*_0_ is the car’s speed during initial contact with the cyclist; *m_a_* is car mass; *m_c_* is the mass of the cyclist and the bicycle.

The full throwing distance of a cyclist and a bicycle after the collision consists of two components, namely the distance travelled in the air after the collision and the distance of sliding on the road surface. However, the movement of a cyclist and bicycle after a collision involves many specifics, including the different nature of contact with the car and the movement after the collision, and characteristics of adhesion to the road surface. Therefore, this movement was further modeled using the computer program PC CRASH 8.1 designed to investigate traffic accidents, which includes the necessary features to perform such modeling.

**Figure 1:**
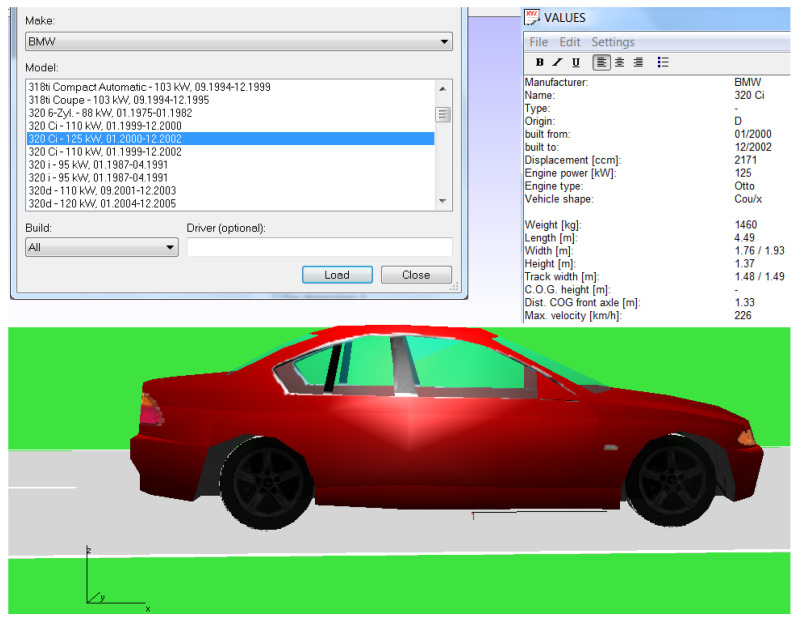
Program PC CRASH 8.1 car model.

The car model used in PC CRASH 8.1 is shown in Figure 1. For modeling using this program, the specific model of the car whose movement is to be modeled, with its corresponding technical characteristics, was first selected from the database. Various car movement modes can be modeled; it is possible to select and evaluate many parameters of the car during modeling, including suspension parameters, degree of loading, type of tires, etc.

**Figure 2:**
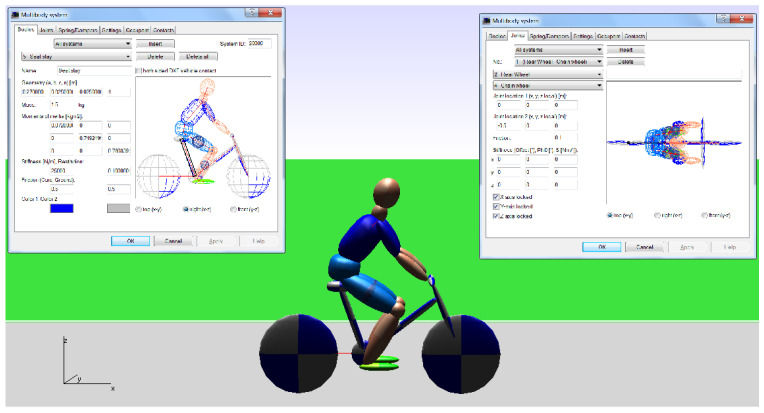
Bicycle and cyclist model.

Figure 2 depicts the cyclist model used in PC CRASH 8.1, modeled in the program as a multi-mass system. Within the software, it is possible to enter and change the masses of individual elements and the parameters of the links between them.

**Figure 3:**
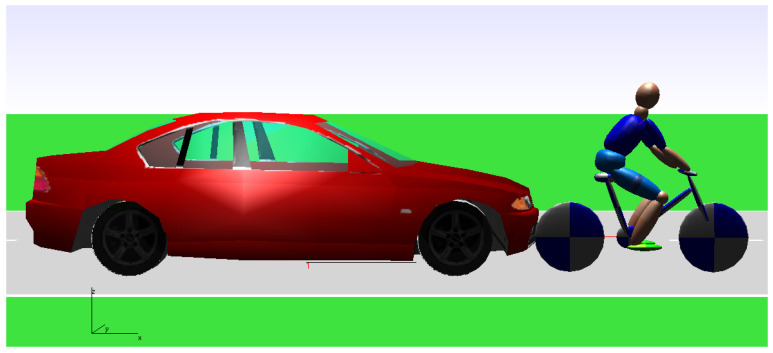
Simulation image of a cyclist being hit, at initial contact between the car and the bicycle.

In this current study, the cycling crash simulation was performed using the program PC CRASH 8.1. Figure 3 shows an image of the modeling at the point of initial contact between the car and the bicycle.

A similar pattern of collision occurs when a car collides with a cyclist at a different angle, i.e., before being hit, the cyclist is not moving parallel to the direction of travel of the car, but at a certain angle. Of course, certain specificities affect the different directions and magnitudes of the forces in such cases. After a collision, the cyclist is usually thrown onto the engine cover of the car and then driven in the direction of travel of the car. If the cyclist is moving in the transverse direction, he is also moved to some extent in his own original direction of travel.

## 3. Research Methodology

The simulations were performed using the computer program PC CRASH 8.1 and the car and cyclist models contained in it.

PC CRASH is software for modeling the movement of vehicles and accident reconstruction; version 8.1 of this program was used for car–cyclist collision simulations in this research. The program allows the coefficient of friction to be set for the whole surface, and afterwards other coefficients of friction can be introduced for separate sections. After the coefficient of friction has been selected, the program automatically selects the maximum possible deceleration of the automobile. It is possible, on the other hand, to introduce the value of deceleration, after which the coefficient of friction must be recalculated. In this case, the road surface for the modeling was assumed to be dry asphalt.

In the modeling using the software PC CRASH 8.1, the definite model of the vehicle was selected from the database, including its appropriate characteristics. Various regimes of movement (braking, acceleration) can be set for the vehicle, including parameters of deceleration and acceleration. It is possible to select and evaluate parameters of the vehicle including suspension, degree of loading, type of the tires, distribution of braking forces, etc. In this case, the simulation assumed that the car was moving at a constant speed before the collision.

During simulation using the computer program PC CRASH 8.1, it was possible to replicate the movement of the car during a traffic accident, and simulate the movement of the vehicle within the space (i.e., in the three planes), taking into consideration such parameters as the profile of the road, including the cross profile, and the location of the vehicle’s center of gravity. This represents is one of the advantages of the PC CRASH 8.1 program, and is important because without this it would be impossible to simulate accidents such as car–cyclist and car–pedestrian collisions. In this case, the simulation assumed that the traffic accident occurred on a horizontal road section.

The cyclist was modeled in the software PC CRASH 8.1 as a multibody system. In this program the cyclist model parameters can be changed, such as the total or individual elements’ mass, for example. More information about the software PC CRASH 8.1, its capabilities, and the models it uses is provided in the operating manual [25].

The simulations were performed for a BMW 320 car and a cyclist. The technical data of the car and the cyclist were taken from the database of the PC CRASH 8.1 computer program. Table 1 presents the data of the car and cyclist used for modeling.

**Figure 4:**
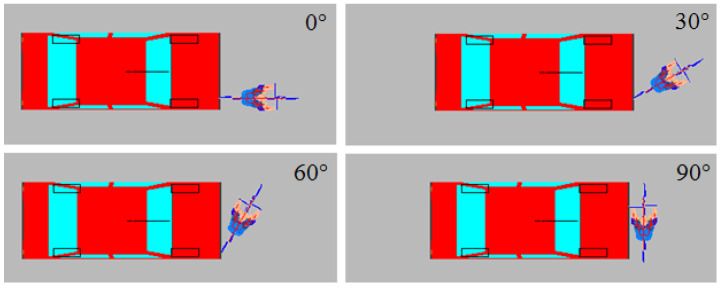
The positions of the car and the cyclist during the impact when the angle of impact wawas 0°, 30°, 60°, or 90°.

Simulation was carried out in multidimensional space. During the simulation, collisions occurred between the car and the cyclist at different angles. These were modeled by accepting the following initial data:

- Vehicle speed from 30 to 90 km/h (at intervals of 10 km/h);
- Cyclist speeds of 10, 15, or 20 km/h;
- Angles of collision between the two vehicles, starting at 0° (car and bicycle moving in the same direction) and ending at 90° (bicycle moving at right angles to the car), changing at intervals of 30° (Figure 4).

Simulations were also performed at other angles between the car and the cyclist (120°, 150°, and 180°). However, these are much less common occurrences in practice, so the results of those tests are not presented in this article.

Figure 4 shows the positions of the car and the cyclist during the impact when the angle of impact was 0°, 30°, 60°, or 90°.

**Figure 5:**
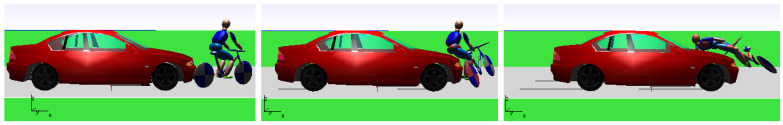
Example of a bicycle collision simulation (collision angle 30°).

Figure 5 shows an example of a cyclist crash simulation with a collision angle of 30°.

When all simulations had been performed, the values of the throwing distance and the throwing angle were compiled and summarized for the cyclist and the bicycle. Based on these data, the respective dependences on the initial speed of the car for the throwing distances and angles of the bicycle and of the cyclist were calculated.

## 4. Research Results Analysis

**Figure 6:**
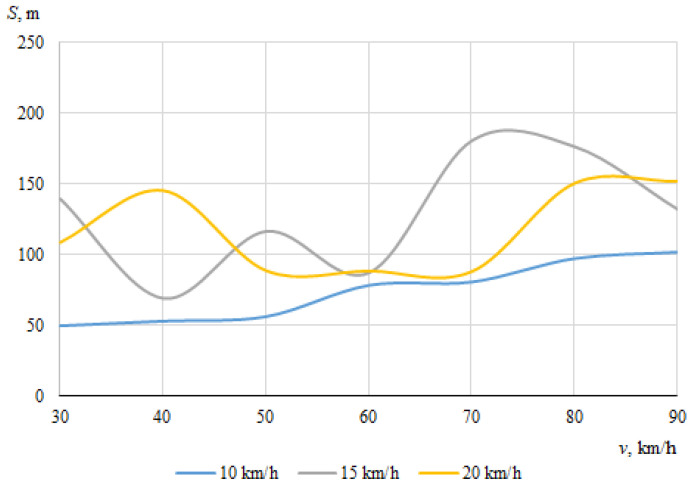
Throwing distances of the bicycle (impact angle 0°).

**Figure 7:**
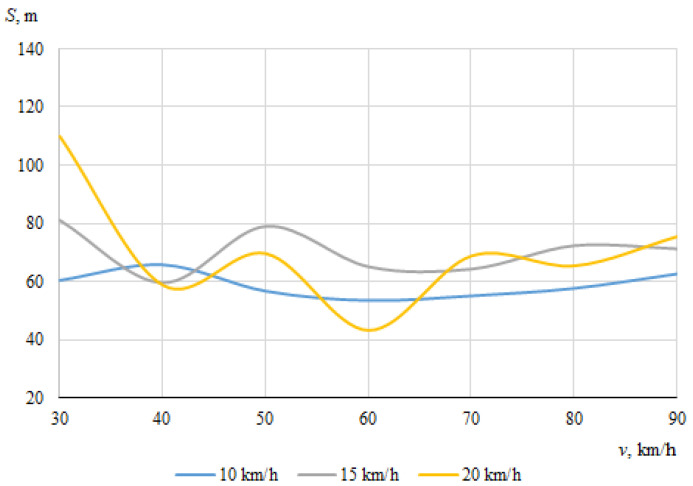
Throwing distances of the cyclist (impact angle 0°).

As described above, the hitting of a cyclist was modeled using different positions of the bicycle in relation to the car, with different angles of collision between the longitudinal axes of symmetry of the car and the cyclist. When the angle between the car and the bicycle was 0° at the time of impact, the speed vectors of the car and the cyclist coincided. In this case, Figure 6 and Figure 7 show the dependences of the throwing distances of the bicycle and the cyclist on the vehicle speeds.

The maximum throwing distance of the cyclist was about 109.7 m, at the lowest car speed (30 km/h) when the speed of the cyclist was 20 km/h. However, it was observed during the simulation that in this case, when the throwing distance of the cyclist was at the maximum, the cyclist moved whilst trapped under the car after impact. It is likely that the throwing distance increased as a result.

The maximum throwing distance of the bicycle was even longer (about 180.4 m), recorded at a car speed of 70 km/h and a cyclist speed of 15 km/h. At a bicycle speed of 10 km/h, it can be seen that as the car speed increased, the throwing distance tendeed to increase (from 49.3 m to 102 m). Generally, however, the bicycle moved chaotically after the impact, sometimes falling under the car and after a while being thrown out from under the car with unpredictable force and angle. After the impact, the bicycle moved according to additional forces caused by the bicycle parts (steering wheel, pedals) rotating at an angular speed.

It can be seen that in this case, when the car and the cyclist were traveling in the same direction before the collision, there was no clear relationship between the throwing distances of the bicycle or the cyclist and the initial speed of the car.

**Figure 8:**
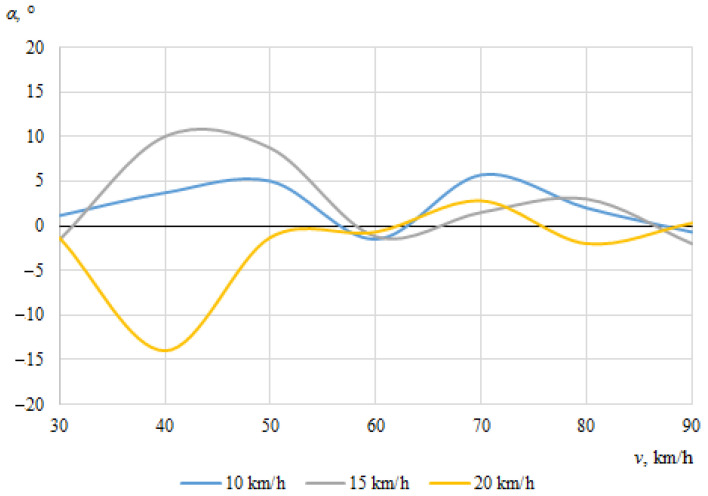
Bicycle throwing angle (impact angle 0°).

**Figure 9:**
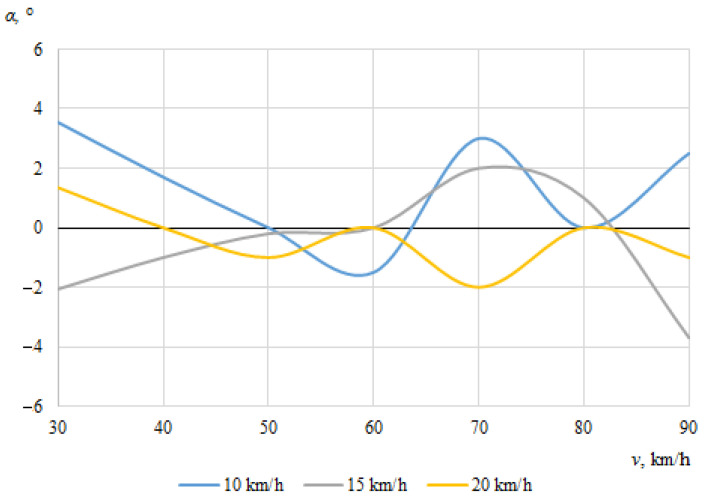
Cyclist throwing angle (impact angle 0°).

The dependences of the throwing angles of the bicycle and the cyclist on the vehicle speeds, when the angle between the cyclist and the car was 0°, are shown in Figure 8 and Figure 9.

It can be seen that the throwing angles of the bicycle and the cyclist were unstable in this case, and changed from one side to another. This can be explained by the fact that when the car and the cyclist were driving in the same direction before the collision, the throw of the bicycle and the cyclist in one direction or another was determined by the above-mentioned random factors as well as instantaneous mass distribution or tilt at the moment of impact.

**Figure 10:**
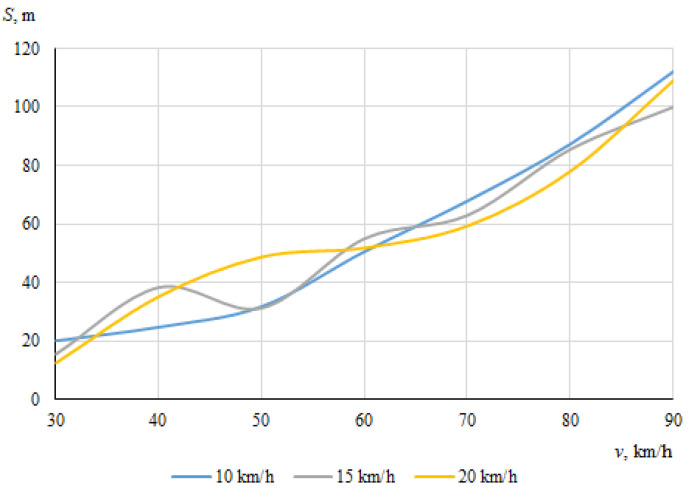
Throwing distances of the bicycle (impact angle 30°).

**Figure 11:**
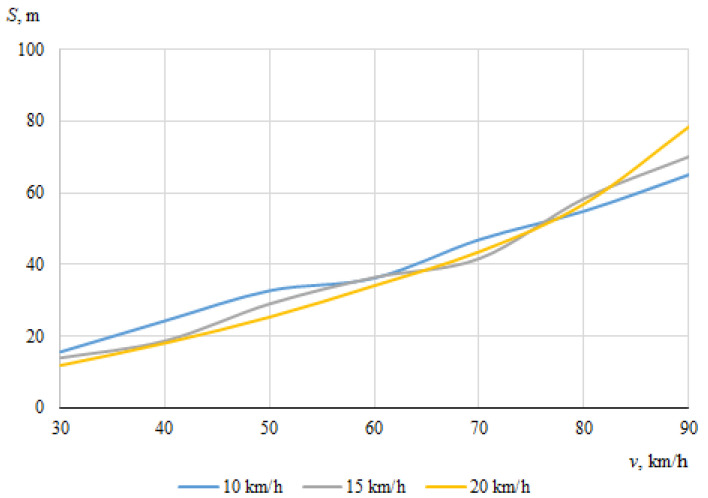
Throwing distances of the cyclist (impact angle 30°).

Figure 10 and Figure 11 show the dependences of the throwing distances of the bicycle and the cyclist on the speeds of the vehicles, when the angle between the car and the bicycle at impact was 30°.

The main trends in this case are already clear; the throwing distance of the bicycle increased as the initial speed of the car increased (Figure 10). However, at a car speed of 50 km/h and a cyclist speed of 15 km/h, the throwing distance of the bicycle suddenly decreased. Such results are not regular, but are determined by random factors on which the movement of a bicycle after a collision with a car depends, such as the specific contact place, the tilt, and the lowering of the bicycle under the car. The minimum throwing distance (12 m) was recorded when the cyclist was traveling at 20 km/h and the car at 30 km/h. The maximum throwing distance for a cyclist (78.4 m) was recorded when the car was traveling at 90 km/h and the cyclist at 20 km/h. Also, at maximum vehicle speeds, it was observed that the displacement of the cyclist in the transverse direction after the impact, with respect to the direction of movement of the car, was significantly greater than at low speeds. In general, it can be seen that as the speed of the car increased, so did the throwing distance of the cyclist (Figure 11).

**Figure 12:**
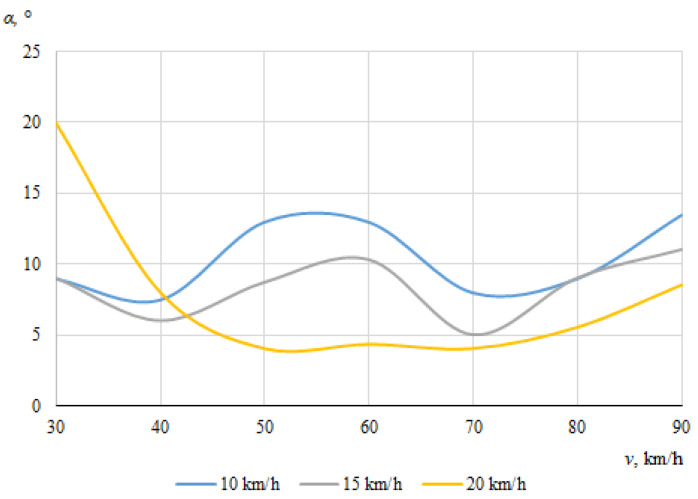
Bicycle throwing angle (impact angle 30°).

**Figure 13:**
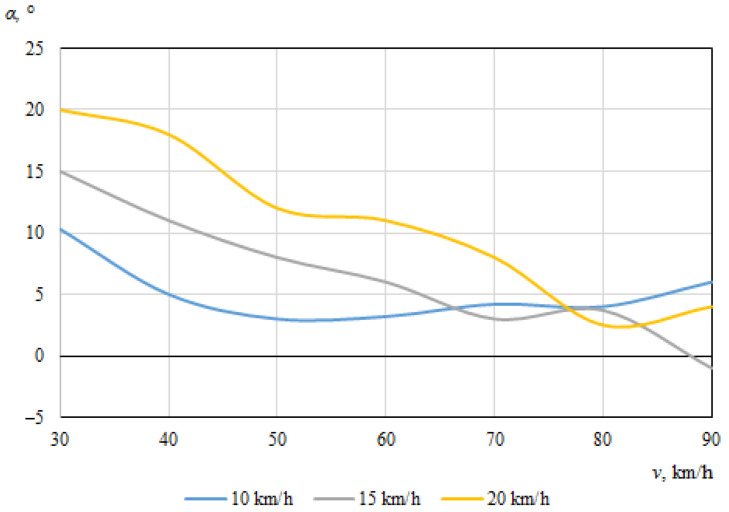
Cyclist throwing angle (impact angle 30°).

Figure 12 and Figure 13 show the dependences of the throwing angles of the bicycle and cyclist on the vehicle speeds, at an angle of 30° during the impact.

No specific trend can be seen, and there does not appear to be a clear relationship between the angle at which the bicycle was thrown and the initial speed of the car (Figure 12); as the bicycle moved chaotically and in some cases fell under the car. As was the case for bicycle throwing distance, this can be explained by various random factors, including the specific contact place of the car and the bicycle, the design of the bicycle itself, the tilting of the bicycle under the car, etc.

Figure 13 shows that the maximum throwing angle of the cyclist was 20°, when the car was moving at its minimum speed of 30 km/h and the cyclist was moving at his maximum speed of 20 km/h. This is because the higher the speed at which the was cyclist riding, the more the cyclist was thrown in the direction of his movement after the impact. When the speed of the car was at its minimum (30 km/h) and the cyclist is also at the minimum (10 km/h), the cyclist was thrown by the impact more in the direction of the car’s travel, meaning that the throwing angle was much smaller, about 10.3°. At the maximum speed of the car (90 km/h), the minimum throwing angle of the cyclist (−1°) was observed. In general, it can be seen that as the speed of the car increased, the throwing angle of the cyclist decreased, as in this case he was thrown more in the car’s original direction of travel after the collision.

**Figure 14:**
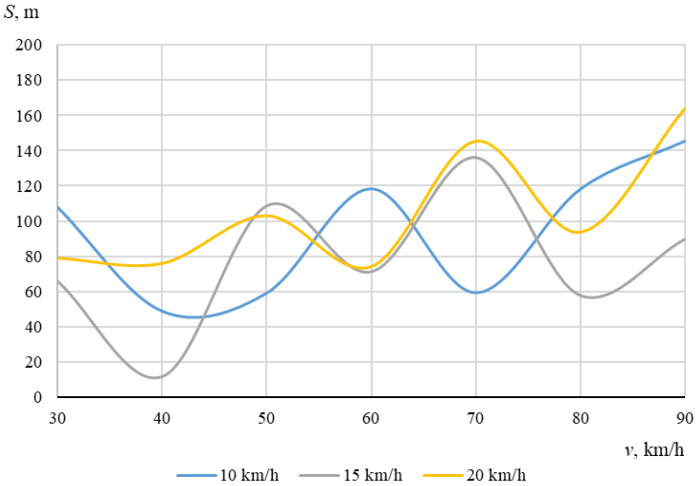
Throwing distances of the bicycle (impact angle 60°).

**Figure 15:**
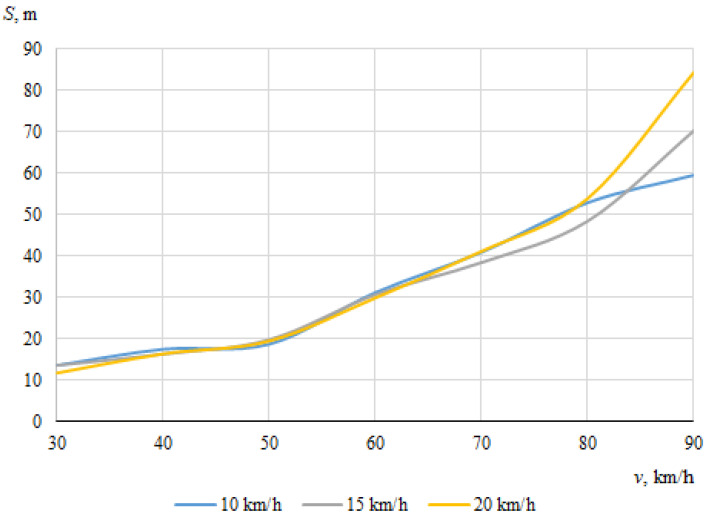
Throwing distances of the cyclist (impact angle 60°).

Figure 14 and Figure 15 show the dependences of the throwing distances of the bicycle and the cyclist on the speeds of the vehicles, when the angle between the car and the bicycle at the time of impact was 60°.

Figure 15 shows the dependence of the cyclist’s throwing distance on the car speed. The maximum throwing distance of 84.4 m was recorded when the car was traveling at a maximum speed of 90 km/h and the cyclist at 20 km/h. At the same car speed, when the cyclist was traveling at 10 km/h, the throwing distance was 59.4 m. This reflects the fact that at higher cyclist speeds, he moved a greater distance in his original direction of travel. The minimum throwing distance of the cyclist wasabout 12 m, recorded at the minimum speed of the car i.e., 30 km/h. There was a clear tendency for the throwing distance of a cyclist to increase as the speed of the car increased (Figure 15).

At an angle of 60° between the car and the bicycle during the crash, there was no clear pattern between the throwing distance of the bicycle and the car’s speed at impact (Figure 14). For each speed of the car there can be seen considerable differences in throwing distances at different speeds of the cyclist. This shows that the bicycle was moving chaotically after the impact. The only visible trend was that as the speed of the car increased, so did the throwing distances of the bicycle.

**Figure 16:**
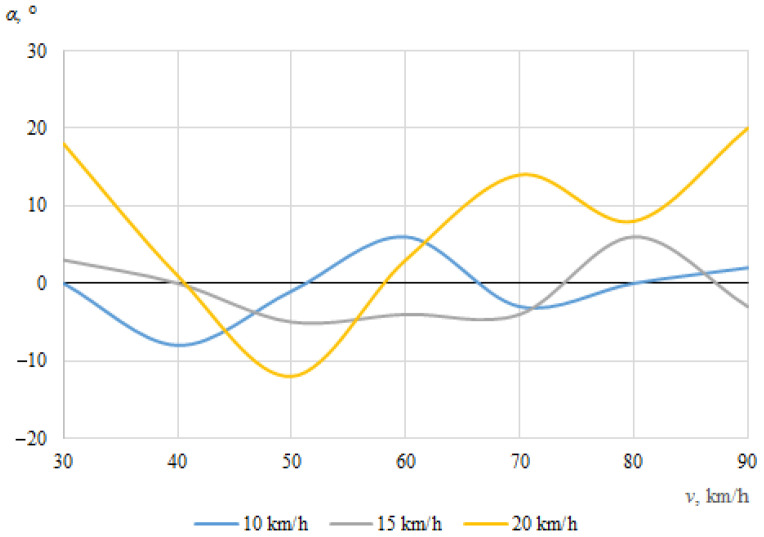
Bicycle throwing angle (impact angle 60°).

**Figure 17:**
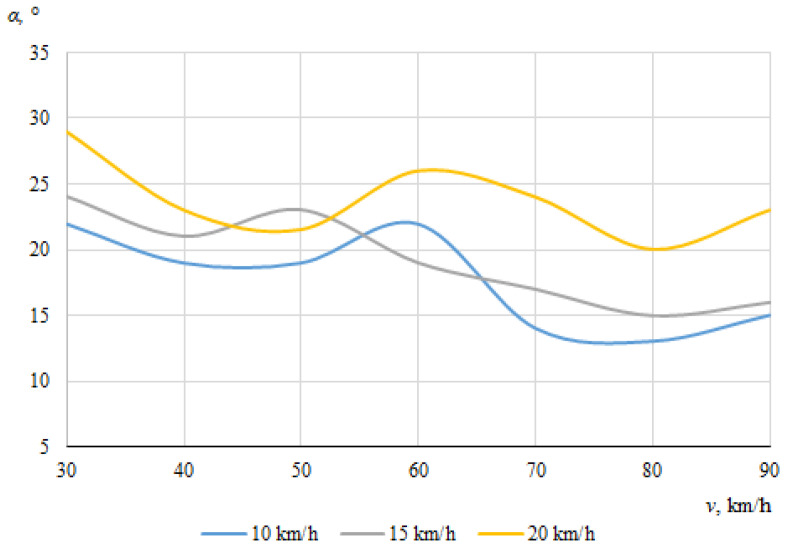
Cyclist throwing angle (impact angle 60°).

Figure 16 and Figure 17 show the dependences of the bicycle and cyclist throwing angles, respectively, on the vehicle speeds when the angle between the car and the bicycle during the impact was 60°.

Figure 17 shows the dependence of the throwing angle of the cyclist on the driving speeds of the vehicles. The maximum throwing angle (29°) for the cyclist was recorded atthe minimum car (30 km/h) and maximum bicycle speed (20 km/h). Due to the small difference in speed between the two vehicles, the cyclist moved more in his original direction of movement after the impact, hence the recorded angle was the largest. The minimum throwing angle of the cyclist was when the car was moving close to its maximum speed and the speed of the cyclist was minimal (10 km/h). These tendencies were similar to those observed for an angle of 30° between car and bicycle at the time of impact. There was a tendency for the throwing angle of the cyclist to decrease as the speed of the car increased, but the decrease in the angle was not very significant.

Figure 16 shows the dependence of the throwing angle of the bicycle on the vehicle speeds. In this case, there was no visible tendency for any clear dependence of the throwing angle of the bicycle on the initial speed of the car. Instead, undulating curves can be observed, indicating that the bicycle was moving chaotically after the impact.

**Figure 18:**
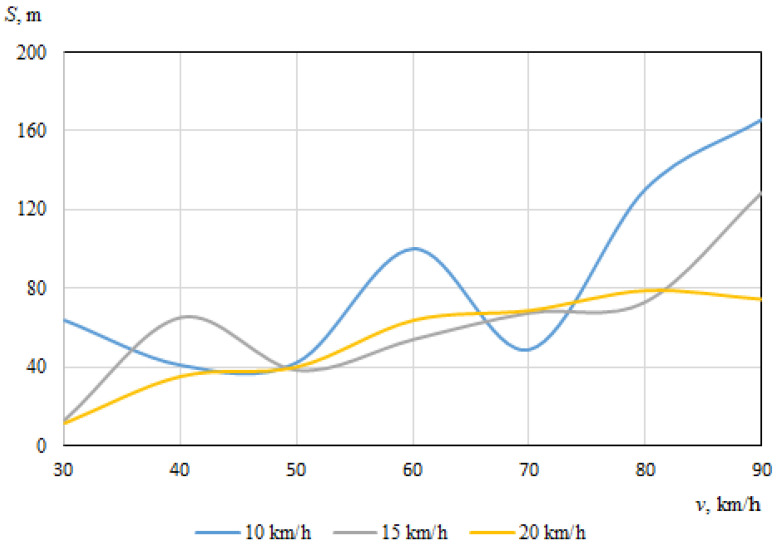
Throwing distances of the bicycle (impact angle 90°).

**Figure 19:**
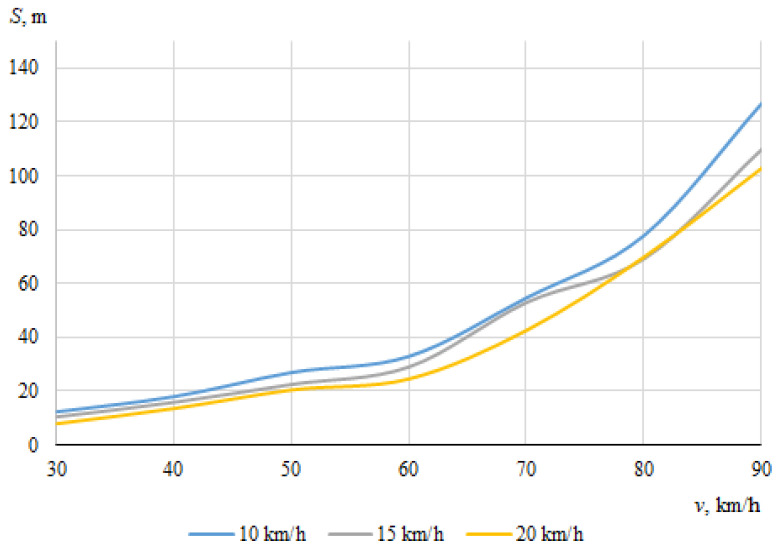
Throwing distances of the cyclist (impact angle 90°).

Figure 18 and Figure 19 show the dependences of the throwing distances of the bicycle and the cyclist on the speeds of the vehicles, when the angle between the car and the bicycle during the impact was 90°.

Figure 19 shows the dependence of the throwing distance of the cyclist on the vehicles’ impact speed. The maximum throwing distance (127 m) was recorded when the car was traveling at 90 km/h and the cyclist at 10 km/h. It can also be seen that across the whole speed range of the car, the cyclist was thrown furthest when he was moving at a speed of 10 km/h, and the difference between the other cyclist speeds was very small. The minimum throwing distance (7.9 m) was recorded when the car was traveling at a minimum speed of 30 km/h before the collision, and the cyclist was traveling at a maximum speed of 20 km/h. A visible pattern shows that as the speed of the car increased, the throwing distance of the cyclist increased. When the car speed was at its minimum 30 km/h, the cyclist slid on the engine cover until he bounced on the windshield. When the car was at its maximum speed of 90 km/h, the cyclist hardly slid on the engine cover during the collision and his head hit the windshield. Due to the low slip and high impulse of force between the car and the cyclist, the latter was thrown over a long distance.

Figure 18 shows the dependence of the throwing distance of the bicycle on the speed of the vehicles. The maximum throwing distance of the bicycle (165.8 m) was recorded when the car was traveling at 90 km/h and the cyclist at 10 km/h. The minimum throwing distance of the bicycle (11.7 m) was recorded when the car was traveling at 30 km/h and the cyclist at his maximum speed of 20 km/h. In general, there was a tendency for the throwing distance of the bicycle to increase as the speed of the car increased, but when the speed of the cyclist was 10 km/h, the bicycle was thrown chaotically. The front of the car made contact with the side of the bicycle, so the higher the speed of the car, the greater the impulse of force, leading to an increased bicycle throwing distance.

**Figure 20:**
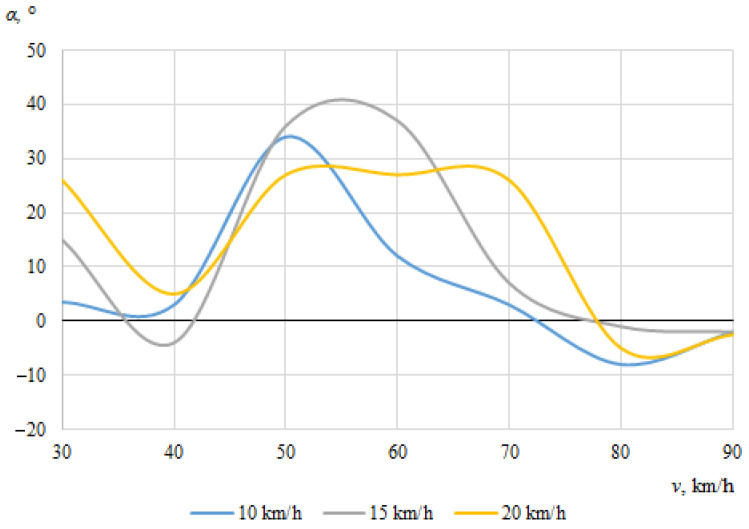
Bicycle throwing angle (impact angle 90°).

**Figure 21:**
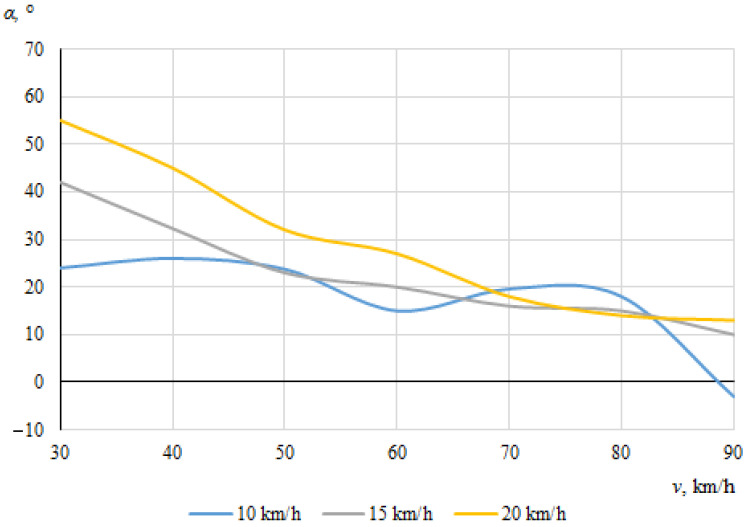
Cyclist throwing angle (impact angle 90°).

Figure 20 and Figure 21 show the dependences on car speed of the throwing angles of the bicycle and the cyclist, respectively, when the angle between the car and the bicycle during impact was 90°.

Figure 21 shows the dependence of the throwing angle of the cyclist on the speeds of the vehicles. The maximum throwing angle (55°) of the cyclist was recorded when the car was traveling at a minimum speed of 30 km/h and the cyclist was traveling at a maximum speed of 20 km/h. When the car was traveling at a maximum speed of 90 km/h and the cyclist at a minimum speed of 10 km/h, the minimum throwing angle (−3°) was recorded. In general, it can be seen that as the speed of the car increased, the throwing angle of the cyclist decreased, as the lower the speed of the car, the more the cyclist moved in his original direction of travel after the impact.

Figure 20 shows the dependence of the throwing angle of the bicycle on the vehicle speeds. The graph shows that the maximum throwing angle of the bicycle was about 40°, when the car was traveling at a speed of 50–60 km/h and the cyclist at a speed of 15 km/h. The minimum throwing angle of the bicycle was recorded when the car was moving at its maximum speed close to 90 km/h. The bicycle moved chaotically over the entire speed range of the car, with significantly lower throwing angles recorded at car speeds of 40 km/h, 80 km/h, and 90 km/h, and higher throwing angle values at 30 km/h, 50 km/h, 60 km/h, and 70 km/h.

Collisions at other angles between the car and the cyclist were also modeled in a similar way, i.e., at 120°, 150°, and 180°. However, these cases are much less common in practice and their results are not presented here.

## 5. Discussion and Conclusions

The analysis of previous studies on this topic shows that in the case of a car–cyclist collision, it is not usually possible to calculate the speed of the car immediately before the traffic accident by applying the methods used in the analysis of car collisions, due to the specifics of individual traffic accidents, the nature of bicycle movement, differences in vehicle masses, etc. However, there are almost no alternative methods for calculating the speed of the car before the traffic accident in the case of a car–cyclist collision. For example, in the case of a car–pedestrian collision, expert studies have sometimes used the methodology of determining the approximate speed of the car based on the distance the pedestrian’s body was thrown after the collision. Results of the current research presented here show that a similar methodology can be developed in the case of a car–cyclist collision. The peculiarity of such an event is that it involves not only a cyclist, but also a bicycle, constituting a multibody system with various natures of contact, and other related variables. In our study, the throwing distances of the cyclist and the bicycle were evaluated, as were the throwing angles, each of which can be used as additional parameters in investigations.

This research has shown that in some cases there is a relationship between the impact speed of the car and both the throwing distance and throwing angle of the cyclist after the accident, in cases of car–cyclist collisions at different collision angles. Because in cases of real traffic accidents, the location of the initial contact is usually known or can be determined, if the final position of the cyclist and the bicycle after the traffic accident is recorded, the full throwing distance and throwing angle of a cyclist can be determined by experts and the impact speed of the car can be evaluated.

In this case, the research was conducted by simulating a car–cyclist collision on a dry asphalt surface on a horizontal road section. Therefore, the research results of this stage can only be applied to traffic accidents that occur in such road conditions. This limits the applicability of the proposed method under other conditions. The selection of the aforementioned conditions was determined by the fact that these are the most commonly encountered in practice.

Future research directions could include refining this methodology and performing more simulations under different road and collision conditions, to create a clear research algorithm for experts to use in various conditions. The use of the summarized results of such simulations would allow experts, when investigating car–cyclist collision cases with knowledge of the throwing distance and angle of the cyclist and the bicycle after the traffic accident, to determine the speed of the car before the impact and perform a more accurate reconstruction of the traffic accident.

Summarizing the results of the conducted research, the following conclusions can be presented:

- By simulating cyclists being hit in different circumstances, i.e., at different speeds and angles of car and cyclist in the event of a collision between the vehicles, using the PC CRASH 8.1 computer program for accident examination, it was found that the throwing angle and usually the throwing distance of the bicycle were not sufficiently informative to determine the speed of the car before the collision, because the movement of a bicycle after such a collision is often chaotic. The bicylce’s movement can depend on many difficult-to-assess circumstances, including the specifics of a particular contact, which are usually not possible to assess, and the possibility of falling under the car.
- When the car and the cyclist were moving in the same direction (i.e., the angle between the travel direction vectors of two vehicles was 0°), the throwing angles and distances of the bicycle and the cyclist were not informative enough to determine the initial speed of the car.
- At an angle of 30°, 60°, or 90° between the longitudinal axes of symmetry of the car and the cyclist, there was a clear relationship between the speed of the car and the throwing distance of the cyclist and, usually, between the speed of the car and the throwing angle of the cyclist.
- The speed of the cyclist did not usually have a significant effect on the throwing distance or the throwing angle enough for it o be taken into account when estimating the initial speed of the car (possibly due to the relatively small range of speeds for the bicycle, which were significantly lower than the carspeeds).
- In certain cases (for example, at an angle of 30°, 60°, or 90° between the longitudinal axes of symmetry of the car and the cyclist), by evaluating the throwing distance and the throwing angle of the cyclist together, with sufficient consistency of results, it is possible to construct a guideline to indicate the speed of the car before hitting the cyclist according to the throwing distance and angle of the cyclist.

## Figures and Tables

**Table 1:** Car and cyclist details.

Car	Model	BMW 320
	Length, m	4.429
	Width, m	1.689
	Height, m	1.328
	Wheelbase, m	2.7
	Front and rear track, m	1.419
	Mass of the car in running order, kg	1316
	Front/rear axles stiffness coefficients, N/m	16,735/15,539
	Front/rear axles damping coefficients, Ns/m	1673/1553
Cyclist	Mass, kg	92
	Bicycle length, m	1.83
	Width with bicycle, m	0.7
	Height with bicycle, m	1.66
	Friction coefficient (cyclist–ground)	0.8
	Friction coefficient (cyclist–car)	0.69

## Data Availability

Not applicable.
